# The Interferon-Induced Protein with Tetratricopeptide Repeats Repress Influenza Virus Infection by Inhibiting Viral RNA Synthesis

**DOI:** 10.3390/v15071412

**Published:** 2023-06-22

**Authors:** Zhengyu Zhu, Xiaoyun Yang, Chaoqun Huang, Lin Liu

**Affiliations:** 1 The Lundberg-Kienlen Lung Biology and Toxicology Laboratory, Department of Physiological Sciences, Oklahoma State University, Stillwater, OK 74078, USA; zhengyu.zhu@okstate.edu (Z.Z.); xiaoyun0775@163.com (X.Y.); chaoqh@okstate.edu (C.H.); 2 Oklahoma Center for Respiratory and Infectious Diseases, Oklahoma State University, Stillwater, OK 74078, USA

**Keywords:** IFIT, influenza virus, viral RNA synthesis

## Abstract

Influenza A virus (IAV) is an eight-segment negative-sense RNA virus and is subjected to gene recombination between strains to form novel strains, which may lead to influenza pandemics. Seasonal influenza occurs annually and causes great losses in public healthcare. In this study, we examined the role of interferon-induced protein with tetratricopeptide repeats 1 and 2 (IFIT1 and IFIT2) in influenza virus infection. Knockdown of IFIT1 or IFIT2 using a lentiviral shRNA increased viral nucleoprotein (NP) and nonstructural protein 1 (NS1) protein levels, as well as progeny virus production in A/Puerto Rico/8/34 H1N1 (PR/8)-infected lung epithelial A549 cells. Overexpression of IFIT1 or IFIT2 reduced viral NP and NS1 RNA and protein levels in PR/8-infected HEK293 cells. Overexpression of IFIT1 or IFIT2 also inhibited influenza virus infection of various H1N1 strains, including PR/8, A/WSN/1933, A/California/07/2009 and A/Oklahoma/3052/2009, as determined by a viral reporter luciferase assay. Furthermore, knockdown of IFIT1 or IFIT2 increased while overexpression of IFIT1 or IFIT2 decreased viral RNA, complementary RNA, and mRNA levels of NP and NS1, as well as viral polymerase activities. Taken together, our results support that both IFIT1 and -2 have anti-influenza virus activities by inhibiting viral RNA synthesis.

## 1. Introduction

Influenza virus belongs to the Orthomyxoviridae family and contains eight negative-sense single-stranded RNA segments [1]. There are four types of influenza virus: A, B, C and D, and only A, B and C cause clinical symptoms in humans [2,3]. Influenza A virus (IAV) is carried by wild aquatic birds as natural hosts [4,5]. In general, IAV is more virulent to humans than influenza B and C viruses. Influenza virus causes an outbreak annually, which is known as flu season [6]. The virus can transmit among the population all year round, but is more active during cold season [7]. Vaccines are the most effective measure to fight against seasonal influenza virus infection [8]. However, influenza virus changes every season, making existing vaccines ineffective, requiring annual updates of flu vaccines [9].

IAV replicates in living cells, especially epithelial cells in the respiratory system. The genome encodes at least eleven proteins. The virus replication cycle starts from hemagglutinin (HA) binding to sialic acid receptors on the cell membrane, followed by the engulfment of the virus into the host cells through endocytosis [10]. Viral ribonucleoproteins (vRNPs) are then released into the cytoplasm. The vRNPs complexes are composed of genomic RNAs, nucleoprotein (NP), polymerase basic protein 1 (PB1), PB2, and polymerase acidic protein (PA). These vRNPs are imported into the nucleus for viral RNA replication and transcription via RNA-dependent RNA polymerase (RdRp) [11,12]. Newly synthesized complementary RNA (cRNA) or mRNA serves as the template for producing new viral RNA (vRNA) or protein. During the late stage of the virus cycle, new vRNPs are exported from the nucleus, assembled with all other viral proteins and finally budded from the cellular surface as progeny viral particles [13].

Interferon (IFN)-induced proteins with tetratricopeptide repeats (IFITs) are IFN-stimulated genes (ISGs) that contain multiple copies of the eponymous tetratricopeptide repeat, a 34-amino-acid helix-turn-helix motif that binds with different protein partners [14,15]. There are four members in humans: IFIT1 (ISG56), IFIT2 (ISG54), IFIT3 (ISG60) and IFIT5 (ISG58), and all of them are clustered on chromosome 10. IFITs are board-spectrum antiviral host factors through their interactions with host proteins and viral RNAs. Multiple mechanisms for IFITs’ antiviral activities against RNA viruses have been proposed [14]: (1) to inhibit the protein translation of viral mRNA via interaction with the eukaryotic initiation factor 3 (eIF3), (2) to bind viral RNA lacking 2’-O methylation to prevent its translation, and (3) to recognize uncapped 5’-ppp of viral RNA and sequester it away from its replication.

IFITs have been shown to inhibit many virus infections including Rabies virus (RABV), Sendai virus (SeV), West Nile virus (WNV), vesicular stomatitis virus (VSV), murine coronavirus, respiratory syncytial virus (RSV), and Hepatitis C virus (HCV) [16,17,18,19,20,21,22,23]. The knockdown of IFIT1, IFIT2, and IFIT3 using siRNA in HeLa cells all increased IAV infection, as measured using a luciferase reporter assay [24], suggesting an antiviral role against IAV. However, knockout of IFIT1 or IFIT2 in human lung epithelial A549 cells had no effects on or even increased IAV infection [25,26].

In this study, we investigated the role of IFIT1 and IFIT2 in influenza virus infection using shRNA knockdown and overexpression approaches and multiple virus assays to examine the underlying mechanisms. We found that IFIT1 and IFIT2 inhibited influenza virus infection and reduced viral RNA synthesis.

## 2. Materials and Methods

### 2.1. Cell Culture

Human lung epithelial A549 cells (American Type Culture Collection, ATCC, Manassas, VA, USA) were cultured in F-12K medium with 10% fetal bovine serum (FBS) and 1% penicillin and streptomycin (PS). Madin–Darby canine kidney (MDCK, ATCC) epithelial cells, human embryonic kidney (HEK) 293, and HEK293T cells (ATCC) were cultured in DMEM containing 10% FBS and 1% PS.

### 2.2. Viruses

H1N1 strains of influenza virus A/Puerto Rico/8/34 (PR/8) and A/WSN/1933 (WSN), and clinical isolates H1N1 A/California/07/2009 (pdm/CA/09) and H1N1 A/Oklahoma/3052/2009 (A/OK/09) were propagated in the allantoic cavities of specific-pathogen-free embryonated chicken eggs (Charles River Laboratories, Wilmington, MA, USA) at 35 °C for 10 days. The allantoic fluids were collected, centrifuged at 2000× *g* for 10 min, and stored at −80 °C. Virus titer was determined using a tissue culture infective dose (TCID_50_) assay [27].

### 2.3. shRNA Vector Construction and Lentivirus Preparation

The IFIT1 and IFIT2 shRNAs were designed using BLOCK-IT^TM^ RNAi designer and inserted into the pmiRZip lentivector (System Biosciences Palo Alto, CA, USA). The primers used for cloning are shown in Table 1. These shRNA vectors were packaged into lentiviruses. Briefly, HEK293T cells were cultured in 10 cm dishes until 80% confluency. The medium was then changed to fresh OPTI-MEM containing 4% FBS. A total of 6 μg of viral packaging (psPAX2), 3 μg of viral envelope (pMD2G), and 1.5 µg of shRNA vectors were mixed in 1 mL OPTI-MEM. Then, 63 µg of PEI was added to the reaction, and it was vortexed and incubated for 15 min in room temperature. The cells were transfected with the mixture for 24 h. The next day, the medium was changed to fresh DMEM containing 10% FBS. The cell culture supernatant was collected after a 48 h culture and then centrifuged at 350 xg for 10 min. The supernatant containing the lentivirus was aliquoted and stored at −80 °C for future use. Viral titers were determined by infecting HEK293T cells with a series of 10-fold dilutions of viral stock in the presence of 4 μg/mL of polybrene. The medium was changed after 24 h infection. Green fluorescent protein (GFP)-positive cells were counted after an additional 48 h culture. Virus titer was calculated based on the following formula, infectious particle/mL = (average of GFP-positive cells from 10 random fields × 594)/(dilution factor × volume of infection) where 594 is fields/well (20X objective) in a 12-well plate.

### 2.4. Knockdown and Overexpression of IFITs

For the knockdown experiments, A549 cells were seeded in a 12-well plate (2 × 10^5^ cells/well) and were then infected with a control shRNA (shCon) or IFIT1/2 shRNA (shIFIT1/2) lentivirus in the presence of 4 μg/mL of polybrene at a multiplicity of infection (MOI) of 100 for 48 h. For the overexpression experiments, HEK293 cells were seeded in a 12-well plate at a density of 5 × 10^5^ cells/well and then transfected with 1.25 µg of pcDNA3.1, pcDNA3.1 3 × Flag IFIT1 or pcDNA3.1 3 × Flag IFIT2 (#53554, #53555, Addgene, Waterton, MA, USA) using Lipofectamine 3000 (Thermo Fisher Scientific, Waltham, MA, USA) for 24 h. Thereafter, the cells were infected with IAV at indicated MOIs in serum-free medium containing 0.5 µg/mL of trypsin treated with L-(tosylamido-2-phenyl) ethyl chloromethyl ketone (TPCK-trypsin, Worthington Biochemical Corporation, Lakewood, NJ, USA) for 1 h. Then, the virus was removed, the medium was changed to fresh SF-DMEM containing 3% bovine serum albumin (BSA), and the cells were cultured for 48 h.

### 2.5. Western Blot Analysis

The cells were lysed with an appropriate volume of T-PER^TM^ Tissue Protein Extraction Reagent (#78510, Thermo Fisher Scientific) containing 1 × Halt^TM^ Protease and Phosphatase Inhibitor Cocktail (#78445, Thermo Fisher Scientific). The protein concentration was determined using the Bio-Rad Protein Assay Kit (Bio-Rad, Hercules, CA, USA). Ten micrograms of each protein sample was electrophoresed on 10% sodium dodecyl-sulfate polyacrylamide gel electrophoresis (SDS-PAGE) gel and transferred onto a nitrocellulose membrane. Western blot analysis was performed using the following primary antibodies and dilutions: mouse anti-NP (HB-65, ATCC, 1:50), mouse anti-non-structural protein 1 (NS1) (#sc-130568, Santa Cruz Biotechnology, Santa Cruz, CA, USA, 1:1000), mouse anti-β-actin (Thermo Fisher Scientific, 1:3000), goat anti-IFIT1 (#PA5-848, Invitrogen, 1:1000), and mouse anti-IFIT2 (#sc-390724, Santa Cruz, 1:1000). The membranes were incubated with primary antibodies overnight at 4 °C, followed by incubation with horseradish peroxidase (HRP)-conjugated goat anti-mouse IgG (Jackson ImmunoResearch, West Grove, PA, USA, 1:2000), or donkey anti-goat IgG (Jackson ImmunoResearch, 1:2000) at room temperature for 1 h. Protein bands were visualized using an enhanced chemiluminescence kit (Thermo Fisher Scientific).

### 2.6. RNA Isolation and Real-Time PCR

Total RNA was extracted using TRI Reagent^®^ (Molecular Research Center, Cincinnati, OH, USA). A total of one µg of RNA was treated with RNase-Free DNase I (Thermo Fihser Scientific) at 37 °C for 30 min and then reverse-transcribed using M-MLV Reverse Transcriptase (Thermo Fisher Scientific) with oligo(dT)18 and random hexamer primers (Promega, Madison, WI, USA). Real-time PCR was carried out in a 20 μL reaction containing specific primers (Table 1) and SYBR Green PCR Master Mix (Eurogentec, Fremont, CA, USA). PCR was performed on ABI 7500 real-time PCR System (Applied Biosystems, Foster City, CA, USA). Cycling conditions for real-time PCR were as follows: 95 °C for 1 min, followed by 40 cycles of 95 °C for 15 s and 60 °C for 1 min. The expression level of a gene was normalized to the internal control, β-actin, and the expression level was calculated using the 2^−ΔCt^ method.

### 2.7. TCID_50_ Assay

The MDCK cells were seeded in 96-well plates at a density of 25,000 cells per well for 24 h. Then, the cells were washed twice with serum-free DMEM. A series of ten-fold dilutions of stocks of allantoic fluid or cell culture supernatant ranging from 10^−1^ to 10^−8^ were prepared in serum-free DMEM medium with 2 μg/mL TPCK-trypsin. One hundred microliters of the diluted virus were added to each well in triplicate. After 72 h of culture, the cells were analyzed for cytopathic effect (CPE), and the TCID_50_ was calculated using the Reed–Muench method [27].

### 2.8. Plaque Assay

The MDCK cells were seeded in 6-well plates at a density of 6 × 10^5^ cells per well. The next day, the cells were washed twice with serum-free DMEM. A series of ten-fold dilutions of stocks of cell culture supernatant ranging from 10^−2^ to 10^−5^ were prepared in serum-free DMEM with 1 μg/mL TPCK-trypsin. The cells were infected with 400 µL of each diluted virus and then incubated at 37 °C for 1 h. The virus solution was aspirated, and the cells were overlaid with medium prepared with 2x DMEM mixing with heated 2% seaplaque agarose (1:1 ratio) containing 1 μg/mL TPCK-trypsin. After the agarose was solidified, the plates were incubated upside down at 37 °C in an incubator for 72 h. The cells were fixed with 10% formalin. The overlay was removed, and the cells were stained with crystal violet and analyzed for plaques. The titer was calculated as plaque number/0.4× dilution factors (PFU/mL).

### 2.9. Immunofluorescence Staining

The cells were fixed in 4% paraformaldehyde for 15 min and permeabilized in 0.2% Triton X-100 for 20 min. Afterwards, the cells were incubated with mouse anti-NP antibodies (HB-65, ATCC, 1:20) in phosphate buffered saline (PBS) containing 10% of normal goat serum for 1 h at 37 °C. The cells were then incubated with Alexa Fluor 546-conjugated goat anti-mouse IgG secondary antibodies (Life technologies, Carlsbad, CA, 1:300) in PBS containing 10% of normal goat serum for 1 h at 37 °C. The cell nuclei were stained with 2 µg/mL of Hoechst 33342 dye (Molecular probes, Waltham, MA, USA). The fluorescence signal was examined with a Nikon fluorescence microscope. The number of NP-positive cells and total number of cells were counted. The infection was presented as the percentages of NP-positive cells over total number of cells.

### 2.10. Luciferase Reporter Assay

To determine the effects of IFIT1 or IFIT2 on different strains of IAV replication, a viral luciferase reporter assay was performed [28]. Briefly, HEK293 cells were transfected with an IAV luciferase reporter vector pHH21-NP-3’-UTR-LUC-NP-5’-UTR (20 ng), pcDNA3.1, pcDNA3.1 3xFlag IFIT1, or pcDNA3.1 3xFlag IFIT2 vector (100 ng) and a pRL-TK vector (2 ng) using Lipofectamine 3000. Twenty-four hours post-transfection, the cells were infected with different strains of IAV, PR/8 (MOI 0.01), WSN (MOI 0.005), pdm/CA/09 (MOI 0.05), and pdm/OK/09 (MOI 0.05) for 48 h. The cells were lysed and dual-luciferase activities were determined using the dual-luciferase reporter assay system (#E1910, Promega, Madison, WI, USA). The reporter activity was expressed as a ratio of firefly to *Renilla* activities, which was further normalized to the control vector.

### 2.11. Viral RNA (vRNA), Complementary RNA (cRNA), and mRNA Quantitation

One µg of RNA was reverse-transcribed using specific primers for each strand and sense: 5’-AGCGAAAGCAGG-3’ and 5’-AGCAAAAGCAGG-3’ for vRNA and 5’-AGTAGAAACAAGG-3’ for cRNA and oligo(dT) for mRNA. Glyceraldehyde-3-phosphate dehydrogenase (GAPDH)-specific primer (5’-GAAGATGGTGATGGGATTTC-3’) was included in all of the reverse transcription reactions for normalization. cDNA was diluted to 1:200 in nuclease-free water. The real-time PCR was performed using the primers listed in Table 1 [29].

### 2.12. Polymerase Activity

A549 cells were seeded in 24-well plates and transfected with 80 ng pCMV3Tag8-NP-FLAG, 40 ng pCMV3Tag8-PB1-FLAG, 40 ng pCMV3Tag8-PB2-FLAG, and 40 ng pCMV3Tag8-PA-FLAG; as well as 200 ng an IAV luciferase reporter vector and 1 ng pRL-TK vector for 24 h. The cells were lysed for dual-luciferase assay.

## 3. Results

### 3.1. IFIT Proteins Inhibit Influenza Virus Replication

**Figure 1:**
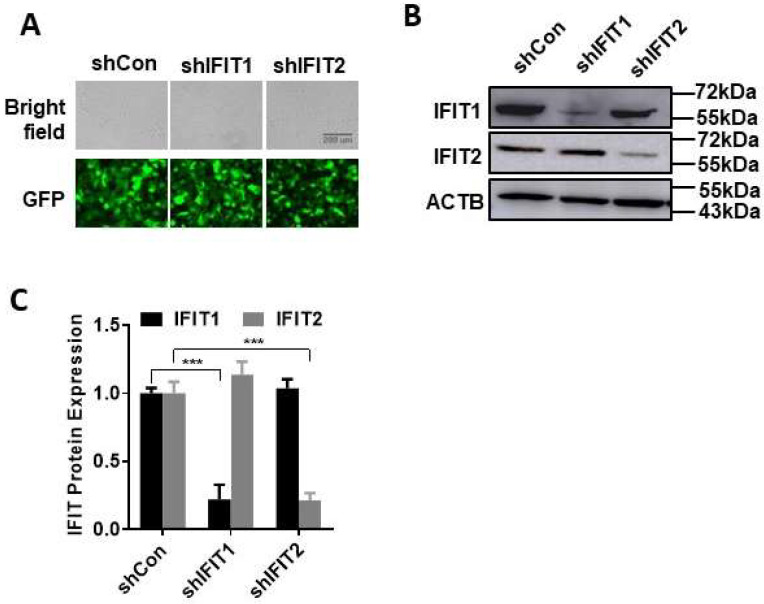
Specific knockdown of IFIT1 and IFIT2 by shRNAs. A549 cells were transduced with the lentiviral shRNA control (shCon), shIFIT1, or shIFIT2 at an MOI of 100 for 48 h and stimulated with 1000 U/mL IFNβ1 for 48 h: (**A**) GFP images; (**B**) representative Western blots; (**C**) quantitation of IFIT1 and IFIT2 protein levels. The results were normalized to β-actin and expressed as a ratio of shCon. Data shown are the means ± SE, *** *p* < 0.001, *n* = 3. Two-way ANOVA, followed by the Tukey test.

To investigate whether IFIT affects influenza virus infection, we used a lentiviral shRNA to knockdown IFIT expression. We first examined the knockdown efficiency and specificity. As the basal expression of IFITs are very low and IFITs are ISGs, we used IFNβ1 to induce IFIT expression. The use of IFNβ1 also avoids the direct effect of a virus on IFIT expression. Human lung epithelial A549 cells were transduced with shIFIT1 or shIFIT2 lentivirus at an MOI of 100 for 48 h before IFNβ1 induction at 1000 U/mL for 24 h. GFP signals showed a high transduction efficiency of shRNA lentivirus (Figure 1A). shIFIT1 reduced the IFIT1 protein levels by 78% ± 0.15 and had no effects on IFIT2 levels. On the other hand, shIFIT2 decreased IFIT2 protein levels by 79% ± 0.08 but did not change IFIT1 protein levels (Figure 1B,C). These results indicate the high knockdown efficiency and specificity of our shRNAs.

**Figure 2:**
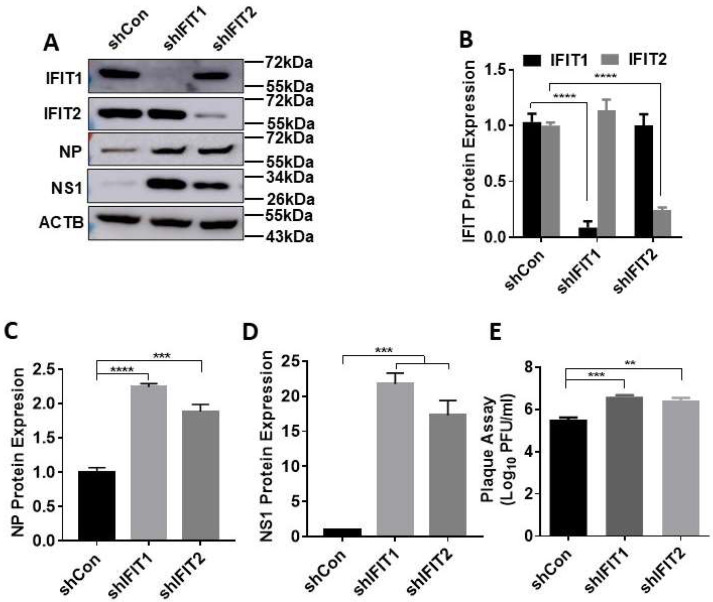
Effects of IFIT1 or IFIT2 knockdown on influenza virus infection in lung epithelial A549 cells. A549 cells were transduced with lentiviral shRNA control (shCon), shIFIT1 or shIFIT2 at an MOI of 100 for 48 h and then infected with PR/8 at an MOI of 0.01 for 48 h. (**A**) The representative western blots. (**B**–**D**) Quantitation of IFIT1, IFIT2, viral NP and NS1. (**C**) Virus titers from culture media. Data shown are means ± SE. ** *p* < 0.01, *** *p* < 0.001, and **** *p* < 0.0001. *n* = 3. One-way (**C**–**E**) and two-way ANOVA (**B**), followed by the Tukey test.

Next, we examined the effects of IFIT knockdown on influenza virus infection. IFIT1 or IFIT2 knocked-down A549 cells were infected with PR/8 at an MOI of 0.01 for 48 h. As in the case of IFNβ1-induced IFIT expression, shIFIT1 and shIFIT2 specifically reduced PR/8-induced IFIT1 and IFIT2 protein levels by 91% ± 0.08 and 75% ± 0.03, respectively (Figure 2A,B). The decrease in IFIT1 or IFIT2 significantly increased viral NP and NS1 protein levels, especially NS1 protein with a 22 ± 2.26- or 17 ± 3.00-fold increase compared to shCon-treated cells (Figure 2A,C,D). Knockdown of IFIT1 or IFIT2 also increased viral titer in culture media approximately by one log as determined by plaque assay (Figure 2E).

**Figure 3:**
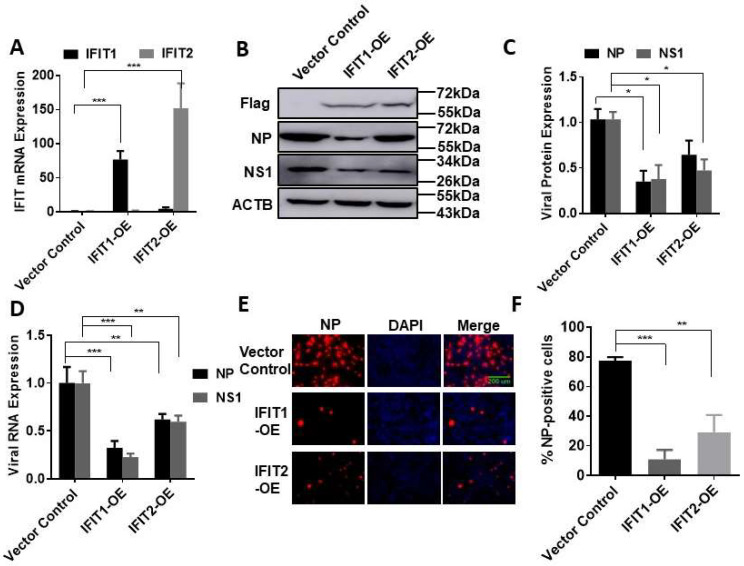
Effects of IFIT1 or IFIT2 overexpression on influenza infection in HEK293 cells: (**A**–**D**) HEK293 cells were transfected with a vector control, Flag-tagged IFIT1 or IFIT2 expression plasmid (IFIT1-OE and IFIT2 OE) for 48 h and then infected with PR/8 at an MOI of 0.01 for 48 h (**A**–**D**) or at an MOI of 5 for 16 h (**E**,**F**). (A) IFIT mRNA levels; (**B**) representative Western blots; (**C**) quantitation of viral NP and NS1 protein levels; (**D**) viral NP and NS1 RNA levels; (**E**) immunofluorescence staining of HEK293 cells with ant-NP antibodies; (**F**) Percentage of NP-positive cells. Data shown are means ± SE. * *p* < 0.05, ** *p* < 0.01, *** *p* < 0.001. *n* = 3. Two-way ANOVA (**A**,**C**,**D**) and one-way ANOVA (**F**), followed by the Tukey test.

To further confirm the effects of IFITs on influenza virus infection, we overexpressed IFIT1 or IFIT2 in HEK293 cells due to the high transfection efficiency in these cells. HEK293 cells were transfected with a Flag-tagged IFIT1 or IFIT2 expression plasmid for 48 h and then infected with PR/8 at an MOI 0.01 for 48 h. Overexpression of IFIT1 or IFIT2 was confirmed at mRNA and protein levels (Figure 3A,B). IFIT1 or IFIT2 overexpression reduced the protein and mRNA levels of NP and NS1 (Figure 3B–D). The effects of IFIT1 or IFIT2 overexpression on influenza virus infection were further demonstrated by immunostaining with anti-NP antibodies. The percentage of the NP-positive cells was 77 ± 2% in the vector control cells and was reduced to 11 ± 6% or 29 ± 11% in the IFIT1- or IFIT2-overexpressed cells (Figure 3E,F).

**Figure 4:**
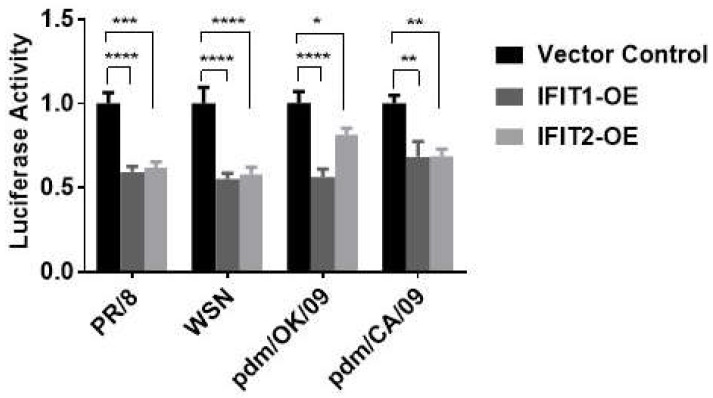
Effects of IFIT1 or IFIT2 overexpression on infection of different strains of influenza virus. HEK293 cells were co-transfected with a vector control, flag-tagged IFIT1 or IFIT2 expression plasmid (IFIT1-OE and IFIT2-OE) together with an IVA luciferase report vector and pRL-TK normalization vector for 48 h, and then infected for 48 h with PR/8 at an MOI of 0.01, WSN at an MOI of 0.005, pdm/OK at an MOI of 0.05, and pdm/CA at an MOI of 0.05. The dual luciferase activities were determined by the ratio of Firefly to *Renilla* activities. Data shown are means ± SE. * *p* < 0.05, ** *p* < 0.01, *** *p* < 0.001 and **** *p* < 0.0001. *n* = 3. Two-way ANOVA, followed by the Tukey test.

To determine whether IFITs had any effects on the infection of other influenza virus strains, we used an influenza virus sensor reporter assay in which a firefly luciferase gene was placed under the control of NP 5’ and 3’ UTRs of influenza WSN [28]. A549 cells were co-transfected with IFIT1 or IFIT2 overexpression or control vector (100 ng), as well as the IAV luciferase reporter vector (NP-UTR-Luc, 20 ng) and a pRL-TK vector (2 ng). Twenty-four hours post-transfection, the cells were infected with commonly used laboratory strains, PR/8 or WSN, and clinical isolates, pdm/CA/09 or pdm/OK/09, for 48 h. IFIT1 or IFIT2 overexpression inhibited all reporter activities (Figure 4), indicating that the antiviral effects of IFIT1 and IFIT2 are independent of influenza virus strains.

### 3.2. IFIT1 and IFIT2 Inhibit Influenza Viral RNA Synthesis

**Figure 5:**
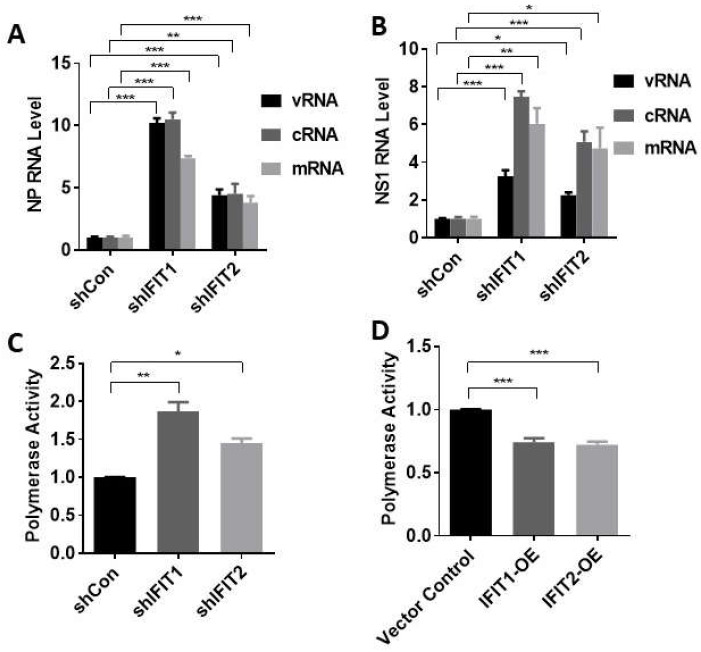
Effects of IFIT1 or IFIT2 knockdown or overexpression on viral RNA synthesis and polymerase activity: (**A**,**B**) Viral RNA synthesis. A549 cells were transduced with lentiviral shRNA control (shCon), shIFIT1 or shIFIT2 at an MOI of 100 for 48 h and then infected with PR/8 at an MOI of 5 for 5 h. NP and NS1 viral RNA (vRNA, cRNA and mRNA) levels were determined. (**C**,**D**) Polymerase activity. HEK293 cells were transfected with shRNA control (shCon), shIFIT1, or shIFIT2 (**C**) or vector control, Flag-tagged IFIT1 or IFIT2 expression plasmid (IFIT1-OE and IFIT2 OE) (**D**) for 24 h, and then co-transfected with minigenome vectors, an IAV luciferase reporter vector and pRL-TK normalization vector for another 24 h. Dual-luciferase activities were determined. Polymerase activity was expressed by the ratio of Firefly to *Renilla* activities and then normalized to shCon (**C**) or vector control (**D**). Data shown are means ± SE. * *p* < 0.05, ** *p* < 0.01, *** *p* < 0.001. *n* = 3. Two-way ANOVA (**A**,**D**) and one-way ANOVA (**C**,**D**), followed by the Tukey test.

To determine how IFITs affect influenza virus infection, we determined the effects of IFIT knockdown on viral RNA synthesis. vRNAs are transcribed into mRNAs, which are translated into viral proteins. vRNA replication requires two steps: vRNAs are first transcribed into cRNA, which serve as templates for new vRNA synthesis [13,30]. The results above have shown that IFIT1 and IFIT2 inhibit viral RNA expression (Figure 3D). However, viral RNAs were measured using cDNAs that were reverse-transcribed from RNAs with oligo(dT) and random hexamer primers, and this method cannot distinguish three types of viral RNAs. We thus measured the levels of vRNA, cRNA, and mRNA using strand- and sense-specific primers for each type of RNA for cDNA synthesis. A549 cells were infected with PR/8 at an MOI of 5 for 5 h for the first cycle of influenza virus replication. We found that all three viral RNAs of NP and NS1 genes increased in IFIT1 or IFIT2-knocked down cells compared to shCon cells (Figure 5A,B).

Influenza viral RNA transcription and replication is mediated by vRNP complexes composed of genomic RNA, RdRp (PB1, PB2, and PA) and its wrapped protein NP. We further determined whether IFIT influences viral polymerase activity using mini-genome assay. Firstly, A549 cells were transfected with shIFIT or IFIT expression vector for 24 h. Then, the cells were co-transfected with NP, PB1, PB2, and PA plasmids together with IAV luciferase reporter vector and pRL-TK normalization vector for another 24 h. The dual luciferase activities, which represent the viral RNA polymerase activity, were measured. The results show that polymerase activities increased in IFIT1- or IFIT2-knocked down cells and decreased in IFIT1- or IFIT2-overexpressed cells (Figure 5C,D). These results suggest that IFIT 1 and IFIT2 inhibit RNA polymerase activity.

## 4. Discussion

Influenza virus, as a zoonotic disease, threats humans, domestic animals, and birds annually. IFITs are the downstream factors of the IFN signaling pathway and have both antiviral and nonviral functions. In this study, we demonstrated that both IFIT1 and IFIT2 are antiviral factors against influenza virus infection and inhibit viral RNA synthesis and polymerase activities.

In general, IFIT genes are widely expressed in many species, including mammals, birds, reptiles, and even fishes. However, the composition and functions of the IFIT family differ among species [14,15]. IFIT genes are lowly expressed under physiological conditions but can be induced by viral or bacterial infection. IFITs are induced by type I and Ⅲ IFNs. IFITs contains the IFN-stimulated response elements (ISRE) in their promoters for the transcription factor complex IFN-stimulated gene factor 3 (ISGF3) to recognize and initiate gene expression. Typically, RNA viruses such as VSV, WNV, and IAV and DNA viruses such as herpes simplex virus and adenovirus primarily engage interferon regulatory factor-3 (IRF-3) or IRF-7 to up-regulate the IFIT expression [15,31,32,33].

In this study, we knocked-down IFIT1 or IFIT2 in lung epithelial cells using lentiviral shRNAs. The specificity of each shRNA was shown by western blot, in which shIFIT1 only knocked down IFIT1, not IFIT2 and vice versa. Knockdown of IFIT1 or IFIT2 increased viral NP and NS1 protein levels and virus particles produced by cells. Furthermore, overexpression of IFIT1 or IFIT2 in HEK293 cells decreased viral mRNA and protein levels, as well as the number of NP-positive cells. These results suggest that IFIT1 and IFIT2 have an anti-influenza virus role, which contradicts with other studies showing that CRISPR knockout of IFIT1 or IFIT2 in A549 cells either had no effects or enhanced IAV infection [25,26]. The reasons for the inconsistent results remain to be determined, but it could be due to the differences in methods used for altering gene expression or assays used for influenza virus infection.

IFITs have been shown to have broad-spectrum antiviral activities [14]. IFIT1 restricts the infection of both RNA and DNA viruses, including RSV, HCV, VSV, Japanese encephalitis virus (JEV), hepatitis B virus (HBV), and others [17,18,24,34,35]. IFITs suppress RSV infection in human cervical carcinoma cells as shown by overexpression of IFIT1, IFIT2 or IFIT3 individually [19]. Transfection of IFIT1 vector into HCV-infected hepatocytes restricts viral RNA replication and virion generation and reduction of IFIT1 levels via RNA interference increases HCV replication [17]. In a mouse study, *Ifit1* deficiency protects mice from VSV infection [24]. IFIT1 is also found to inhibit the replication of JEV [34] and WNV [18]. Another study shows that silencing of IFIT1 increases HBV replication in hepatoma cells [35].

IFIT2 has also been reported to be antiviral against RABV, WNV, VSV, SeV, and murine coronavirus [20,21,22,23,24]. In mouse neuroblastoma cells, IFIT2 silencing by siRNA increases the number of viral RNA copies in a time-dependent manner and *Ifit2^-/-^* mice have an increased mortality after RABV infection [20]. In vivo knockout mice studies show that *Ifit2* is required for controlling WNV infection in brain [21]. *Ifit2^-/-^* mice are also more susceptible to VSV and SeV infections compared to wild type mice [21,22,23,36]. Similar results were found in murine coronavirus infection, in which the deficiency of *Ifit2* increases clinical scores and viral titers within a 7-day infection [23].

Our current studies showed that knockdown of IFIT1 and IFIT2 increased the expression levels of all three types of viral RNAs, vRNA, cRNA, and mRNA, suggesting that IFIT1 and IFIT2 inhibited viral genome transcription and replication. This effect is likely mediated by their effects on RdRp, as IFIT1 and IFIT2 also inhibited viral polymerase activity as measured by a minigenome assay. This result is consistent with a previous study showing that siRNA knockdown of IFIT1 or IFIT2 reduces vRNA expression in HeLa cells as demonstrated by a luciferase reporter assay [24]. However, how IFITs regulate RdRp remains to be determined. One of the mechanisms could be the binding effect of IFITs to viral mRNAs, including 5’ capped 2’-O unmethylated mRNA and 5’-triphosphate RNA (PPP-RNA) [24,32,34]. Another mechanism could be because of the translation inhibition via their binding to eIF3.

In conclusion, IFIT1 and IFIT2 are anti-influenza proteins through their inhibitory effects on viral RNA synthesis.

## Figures and Tables

**Table 1:** Primers for real-time PCR and constructing shRNA vectors.

Primer	Forward	Reverse
Human IFIT1	GCGCTGGGTATGCGATCTC	CAGCCTGCCTTAGGGGAAG
Human IFIT2	GACACGGTTAAAGTGTGGAGG	TCCAGACGGTAGCTTGCTATT
NP	TGTGTATGGACCTGCCGTAGC	CCATCCACACCAGTTGACTCTTG
NS1	CCGACATGACTCTTGAGGAAAT	CGCCTGGTCCATTCTGATAC
IFIT1-shRNA	GATCCGGAAGAACATGACAACCAAGCTTCAAGAGAGCTTGGTTGTCATGTTCTTCCTTTTTG	AATTCAAAAAGGAAGAACATGACAACCAAGCTCTCTTGAAGCTTGGTTGTCATGTTCTTCCG
IFIT2-shRNA	GATCCGCCCTGGAATGCTTACGTAAATTCAAGAGATTTACGTAAGCATTCCAGGGCTTTTTG	AATTCAAAAAGCCCTGGAATGCTTACGTAAATCTCTTGAATTTACGTAAGCATTCCAGGGCG

## Data Availability

The data presented in this study are available in this article.
